# iNOS Associates With Poor Survival in Melanoma: A Role for Nitric Oxide in the PI3K-AKT Pathway Stimulation and PTEN S-Nitrosylation

**DOI:** 10.3389/fonc.2021.631766

**Published:** 2021-02-12

**Authors:** Zhen Ding, Dai Ogata, Jason Roszik, Yong Qin, Sun-Hee Kim, Michael T. Tetzlaff, Alexander J. Lazar, Michael A. Davies, Suhendan Ekmekcioglu, Elizabeth A. Grimm

**Affiliations:** ^1^ Department of Melanoma Medical Oncology, The University of Texas MD Anderson Cancer Center, Houston, TX, United States; ^2^ Department of Dermatologic Oncology, National Cancer Center Hospital, Tokyo, Japan; ^3^ Department of Genomic Medicine, The University of Texas MD Anderson Cancer Center, Houston, TX, United States; ^4^ Department of Pharmaceutical Sciences, School of Pharmacy, The University of Texas at El Paso, El Paso, TX, United States; ^5^ Department of Dermatopathology and Oral Pathology, University of California San Francisco, San Francisco, CA, United States; ^6^ Department of Pathology, The University of Texas MD Anderson Cancer Center, Houston, TX, United States

**Keywords:** melanoma, phosphatase and tensin homolog, nitric oxide, PI3K-AKT axis, S-nitrosylation, inducible nitric oxide synthase, AKT activation

## Abstract

We previously showed that inducible nitric oxide synthase (iNOS) protein expression in melanoma tumor cells is associated with poor patient prognosis. Here, we analyzed the association between iNOS and the oncogenic PI3K-AKT pathway. TCGA data show that iNOS and phospho-Akt Ser473 expression were associated significantly only in the subset of tumors with genetically intact PTEN. Employing a stage III melanoma TMA, we showed that iNOS protein presence is significantly associated with shorter survival only in tumors with PTEN protein expression. These findings led to our hypothesis that the iNOS product, nitric oxide (NO), suppresses the function of PTEN and stimulates PI3K-Akt activation. Melanoma cells in response to NO exposure *in vitro* exhibited enhanced AKT kinase activity and substrate phosphorylation, as well as attenuated PTEN phosphatase activity. Biochemical analysis showed that NO exposure resulted in a post-translationally modified S-Nitrosylation (SNO) PTEN, which was also found in cells expressing iNOS. Our findings provide evidence that NO-rich cancers may exhibit AKT activation due to post-translational inactivation of PTEN. This unique activation of oncogenic pathway under nitrosative stress may contribute to the pathogenesis of iNOS in melanoma. **Significance:** Our study shows that iNOS expression is associated with increased PI3K-AKT signaling and worse clinical outcomes in melanoma patients with wt (intact) PTEN. Mutated PTEN is already inactivated. We also demonstrate that NO activates the PI3K-AKT pathway by suppressing PTEN suppressor function concurrent with the formation of PTEN-SNO. This discovery provides insight into the consequences of inflammatory NO produced in human melanoma and microenvironmental cells. It suggests that NO–driven modification provides a marker of PTEN inactivation, and represents a plausible mechanism of tumor suppressor inactivation in iNOS expressing subset of cancers.

## Introduction

Melanoma is the deadliest form of skin cancer and its incidence has been increasing in the United States for the last 30 years. The molecular pathogenesis of melanoma is strongly associated with activation of the phosphatidylinositol 3-kinase (PI3K) –AKT pathway, promoting cellular growth, proliferation, and survival. In melanoma, activation of the PI3K-AKT pathway generally occurs in the setting of concurrent oncogenic RAS-RAF-MEK-ERK signaling (1). Previous studies showed that the constitutive activation of the PI3K-AKT pathway occurred in the setting of PTEN loss of expression and function, which was detected in up to 30% of cutaneous melanomas (2). The analyses of human melanoma samples and cell lines showed that low PTEN levels were associated with elevated phospho(active)-AKT, which remained significant even in brain metastasis (3). Supporting the clinical significance of activation of the PI3K-AKT pathway, PTEN loss was shown to promote resistance to both targeted and immune therapies for melanoma (4).

In addition to genetic alterations, PTEN function can be regulated by post-translational modifications (PTM) (5). Lee et al. have shown that cysteines at the active site of PTEN normally form a disulfide bond, and oxidation by H_2_0_2_ exposure results in its inactivation (6). More recently, cysteines of PTEN were reported to be post-translationally modified by another oxidant, nitric oxide (NO) resulting in S-Nitrosylation (SNO), in human brain, which was indicated to promote neuron survival in early stages of neurodegenerative disease (7). We have previously reported that protein expression of nitric oxide synthase (NOS), particularly inducible NOS (iNOS), in approximately 60% of tumor cells in the advanced melanoma patients, and that its presence significantly associated with poor prognosis (8). It has also been proposed that cancer-associated inflammation and NO production support cancer by oxidizing redox-sensitive molecules, especially tumor suppressors (9). In this study, we report that iNOS expression is associated with increased PI3K-AKT signaling and worse clinical outcomes in melanoma patients with intact PTEN, which this is possibly due to iNOS-driven oxidants. We demonstrate that the iNOS product, NO, can activate the PI3K-AKT pathway by suppressing PTEN activity concurrent with the formation of PTEN-SNO. Together the findings provide new insights into the significance and multifaceted roles of NO in melanoma and provide evidence for PTM regulation of tumor suppressors.

## Materials and Methods

### Materials

DETA NONOate (#82120), 1400W (#80200), and L-NIL (#80300) were purchased from Cayman Chemical (Ann Arbor, MI). Sulfanilamide (#S9251), Sodium Nitrite (#S2252), N-(1-Naphthyl) ethylenediamine dihydrochloride (#22,248-8), and sodium ascorbate (#A7631) were purchased from Sigma-Aldrich (Saint Louis, MO). N-Ethylmaleimide (NEM) (#23030), IP Lysis Buffer (#87787), Protease and Phosphatase Inhibitor Cocktail (#78440), protein quantification assay kit (#A53226) Streptavidin Magnetic beads (#65602), N-[6-(biotinamido) hexyl]-3′-(2′-pyridyldithio)-propionamide (HPDP-Biotin) (#21341) and Protein A Agarose Beads (#20333) were purchased from Thermo Fisher Scientific (Waltham, MA.). LY294002 (#S1105) and Wortmannin (#2758) were purchased from Selleck Chemicals (Houston, TX). All of the cell culture media was purchased from Corning (Corning, NY). All antibodies, including phospho-AKT Ser 473 (pAKTS473)(#4060), AKT (#9272), PTEN (#9559), GSK-3α/β (#5676), phospho-GSK-3α/β (#8566), and PTEN (#9788), were purchased from Cell Signaling Technology (Beverly, CA) unless stated otherwise. We purchased iNOS antibody (#SC-651) from Santa Cruz Biotechnology (Santa Cruz, CA).

### Melanoma Tissue Microarrays

Slides from a stage III melanoma TMA (Stage III B/C array of regional metastases) were obtained from MD Anderson Cancer Center (4). Immunolabeling of PTEN (2) and iNOS were assessed and scored using standard methods as previously described (8). This study was approved by the institutional review boards of MD Anderson Cancer Center and complied with the 1983 revision of the Helsinki Declaration of 1975.

### Cell Culture, Treatment, and Transfection

The human melanoma cell lines A375, MeWo, LOX IMVI, and WM793 were obtained from the American Type Culture Collection (ATCC), and WM 266.4 was provided by Dr. Michael Davies (MD Anderson). Cells were cultured in Dulbecco’s modified Eagle medium containing 5% fetal bovine serum at 37°C and in 5% CO2 and 95% air. For DETA NONOate treatment, cells were cultured to 75% confluency, and freshly prepared DETA NONOate stock was added to the final concentration as indicated in the text. After treatment times, cells were washed twice with cold phosphate-buffered saline and were harvested for analysis of pAKTS473 levels, AKT kinase activity, AKT substrate phosphorylation, and PTEN activity. An iNOS expressing construct was established as described by us previously (10). Cells were transfected with plasmids using Lipofectamine 2000 (Invitrogen), according to the manufacturer’s specifications.

### *In Vitro* Akt Kinase Assay

*In vitro* Akt activity was assessed by using an Akt kinase assay kit (#9840, Cell signaling Technology). After DETA NONOate treatment, A375 cells were lysed, and cellular Akt was immunoprecipitated using beads containing immobilized Phospho-AKT (S473) Rabbit monoclonal antibody. The kinase reaction was performed by supplementing the kinase substrate GSK-3 fusion protein and ATP. The phospho-GSK3 was measured by Western blot using an anti-phosho-GSK-3 antibody.

#### Akt Signaling Pathway Antibody Array

The phosphorylation of 16 effector proteins associated with the Akt signaling, were screened by an Akt signaling antibody array kit (#9474, Cell Signaling Technology) according to the manufacturer’s instructions. Briefly, cell lysates were incubated on the glass slides containing target-specific capture antibodies followed by a biotinylated detection and chemiluminescence visualization.

### Western Blotting

After DETA NONOate treatment, cells were washed with cold PBS twice and lysed with IP lysis buffer [25 mM Tris•HCl pH 7.4, 150 mM NaCl, 1% NP-40, 1 mM ethylenediaminetetraacetic acid (EDTA), 5% glycerol] and a protease and phosphatase inhibitor cocktail. Protein concentration was determined by bicinchoninic acid (BCA) assay. Equal amounts of protein were separated with sodium dodecyl sulfate-polyacrylamide gel electrophoresis (SDS-PAGE) transferred to a nitrocellulose membrane and blocked in 5% bovine serum albumin in phosphate-buffered saline/Tween. The membrane was then incubated with primary and secondary antibodies, and target proteins were detected and visualized with enhanced chemiluminescence (ECL) detection reagent.

#### Biotin-Switch Assay for SNO-Post Translational Modification Detection

The biotin-switch assay for detection of SNO-modifications on thiols was performed as described previously (11) with minor modifications. In brief, cells expressing iNOS were lysed in HENS buffer (100 mM HEPES, 1 mM EDTA, 0.1 mM neocuproine, 1% Triton X-100, 0.1% SDS, pH 7.4), and free thiols were blocked by incubation in 50 mM NEM. Protein SNOs were selectively reduced by ascorbate (20 mM) and labeled with HPDP-Biotin (1 mM). The biotinylated proteins were collected on streptavidin magnetic beads and eluted for immunoblotting analysis using antibody specific for PTEN.

### PTEN Phosphatase Activity Assay

PTEN phosphatase activity was assessed by measurement of inorganic phosphate liberated during *in vitro* PTEN phosphatase reactions converting PIP_3_ to PIP_2_. Briefly, after DETA NONOate treatment, A375 cells were lysed, and cellular PTEN was immunoprecipitated and added to assay buffer containing substrate phosphatidylinositol 3,4,5-trisphosphate diC8 [PI(3,4,5)P_3_] (#P3908, Echelon Biosciences, Salt Lake City, UT). Free phosphate was detected using a Malachite Green detection kit (#10009325, Cayman Chemical) according to the manufacturer’s instruction.

### Nitrite Measurements

The nitrite level in the medium was measured based on the Griess reaction. Briefly, 200 μl of the cell culture medium was mixed with 10 μl of sulfanilamide (30 mM in 2 M HCl), followed by 10 μl of N-(1-naphthyl)ethylenediamine dihydrochloride (30 mM in 0.1 M HCl). The absorbance was measured at 540 nm and compared with a standard curve generated using sodium nitrite.

### Statistical Analysis

Statistical results are reported as means ± standard deviations for n ≥ 3 biological replicates. Statistical analysis was performed using GraphPad Prism 7.0, and significance was evaluated with a two-tailed Student t-test. For the survival analysis, patients who remained alive at the time the study data were collected were censored at the date of their last follow-up. The Kaplan-Meier method was used to estimate the distribution of survival times, and the log-rank test was used to compare distributions. For the reverse phase protein array (RPPA) protein expression analysis, the Mann-Whitney test was used, and a difference was considered significant for a p value <0.05.

The Cancer Genome Atlas (TCGA) gene expression, the Reverse Phase Protein Array (RPPA) protein expression, and clinical data were downloaded from official TCGA repositories (Broad Institute, TCGA Genome Data Analysis Center). RPPA box plots were prepared using the Tableau Desktop software.

## Results

### iNOS Expression Is Associated With Increased AKT Activity and Poor Clinical Outcomes in Melanomas With Intact PTEN

**Figure 1:**
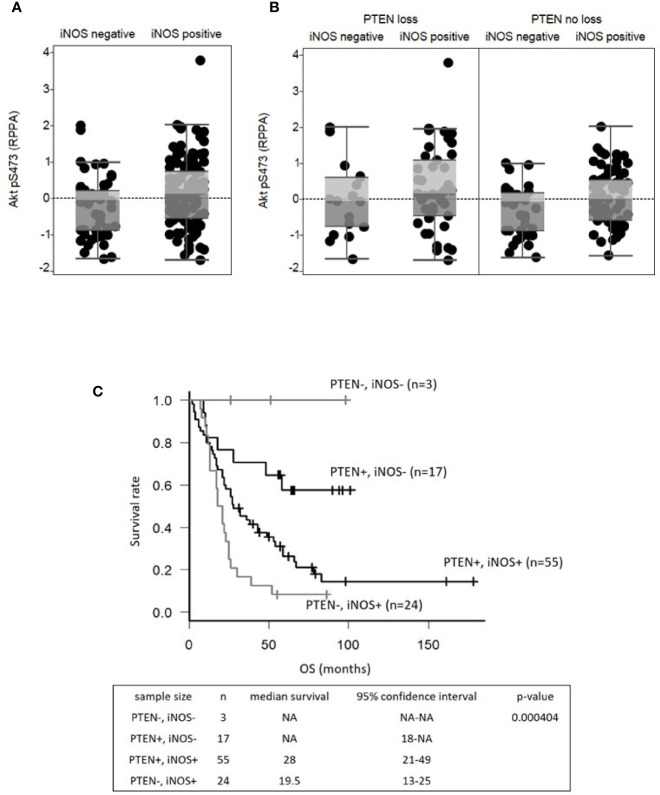
iNOS expression is associated with increased AKT activity and poor clinical outcomes in melanomas with intact PTEN. Reverse phase protein array analyses show **(A)** expression levels of the phospho-AKT Ser 473 protein (pAKTS473) in iNOS-negative (<0.1 transcripts per kilobase million) and iNOS-positive (≥ 0.1 transcripts per kilobase million) tumors in the melanoma TCGA, and **(B)** pAKTS473 protein expression levels in iNOS-negative and iNOS-positive groups with PTEN loss (mutated PTEN or PTEN copy number < -0.5) and without PTEN loss. A P value <0.05 was considered statistically significant. **(C)** Kaplan-Meier survival analysis based on combined markers of PTEN and iNOS status in tumor cells in stage III melanoma patients. A P value <0.05 was considered statistically significant. OS, overall survival.

To examine the association between iNOS and AKT activity in clinical samples, we first analyzed the RPPA data of melanoma in TCGA (12) to quantitatively measure Phospho-Akt S473 (pAKTS473) levels. As pAKTS473 is a marker of Akt activation, the levels of expression were compared between tumors with or without positive iNOS gene expression (>= 0.1 TPM by RNAseq). The analysis identified significantly (p < 0.05) higher pAKTS473 expression levels in tumors with positive iNOS gene expression (n=112, median = 0.114) versus those without iNOS gene expression (n=57, median = -0.0451) (**Figure 1A**).

As loss of PTEN expression is also associated with increased pAKTS473 expression, we then repeated the analysis incorporating the genetic status of PTEN (PTEN loss for mutation or copy number < -0.5 vs. PTEN no loss). The data show that in tumors with PTEN loss, there was no significant difference in pAKT expression (p=0.332) as a function of iNOS. However, in tumors with genetically intact PTEN, iNOS gene expression was associated with significantly increased pAKTS473 expression (p<0.05; iNOS negative: n=40, median expression = -0.0638, iNOS positive: n=71, median expression = 0.0) (**Figure 1B**). These results led us to hypothesize that iNOS may be involved in AKT activation in the subset of melanomas with functionally active intact PTEN.

The prognostic significance of iNOS in melanomas with intact PTEN was further analyzed using a TMA of lymph node metastases from patients with newly diagnosed stage III disease (8). Previous analysis showed that loss of PTEN protein expression was associated with significantly shorter overall survival (OS) among these patients (4). Based on the associations observed in the melanoma TCGA cohort, we tested whether iNOS protein expression was associated with clinical outcomes among stage III patients with intact PTEN expression. iNOS protein levels were determined by IHC, which was categorized as positive or negative based on the percentage of positively stained cells. Among the tumors with PTEN protein expression, iNOS was positive in 55 (76.4%) and negative in 17 (23.6). Kaplan-Meier analysis showed that patients with positive iNOS expression had significantly shorter OS (median 28.0 months) from stage III diagnosis compared to patients with negative iNOS (median OS not reached, p=0.012) (**Figure 1C**). Most patients with negative PTEN expression were iNOS positive (24 vs. 3) in this TMA (TMA core examples in **Supplementary Figure 1**).

### Nitric Oxide Activates PI3K and AKT in Human Melanoma Cells With Intact PTEN

**Figure 2:**
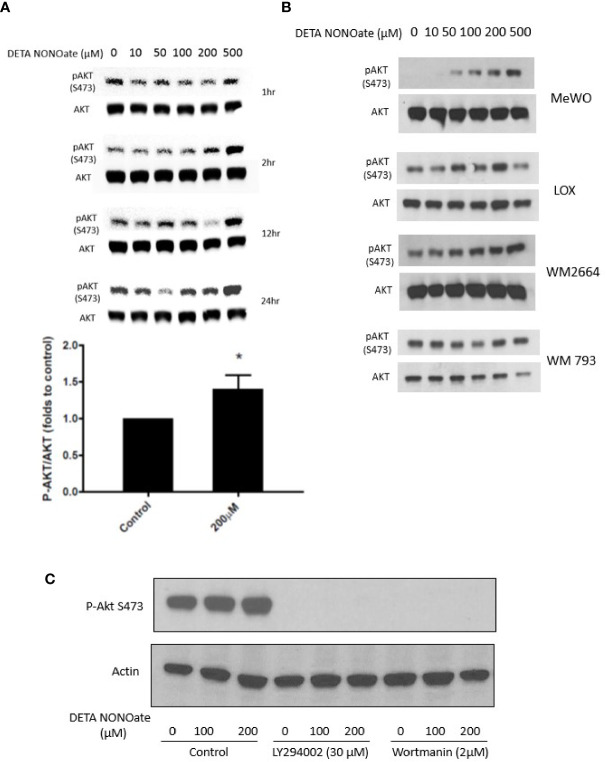
Nitric oxide stimulates PI3K and activates AKT in melanoma cells. **(A)** A375 cells were treated with NO donor DETA NONOate (0–500 µM) for 1, 2, 12, and 24 h. P-Akt (S473) levels were examined by western blot. At 2 h, 200 µM DETA NONOate treatment significantly increased P-AKT (S473) level. **(B)** MeWo, LOX, WM 266-4, and WM 793 cells were treated with DETA NONOate (0-500 µM) for 2 h P-AKT (S473) expression was examined by western blot. **(C)** PI3K inhibitor LY294002 and Wortmann pretreatment both blocked basal and NO-induced P-AKT(S473) formation.

Based on the above clinical associations, we performed further experiments to functionally evaluate the relationship between iNOS, PTEN, and the PI3K-AKT pathway in human melanoma cell lines. First, A375, a human melanoma cell line with a BRAF V600E mutation and wild type PTEN, was exposed to the NO donor, DETA NONOate for defined times up to 24 h. It has been shown previously that DETA NONOate addition to cells generates intracellular NO flux at approximately 1000:1 ratio. Thus, µM concentration of DETA NONOate result in a steady state of NO at nM concentrations, which is responsible for its biological function (13). Western blotting showed that NO exposure increased the level of pAKT S473 in the cells within 2 h, which was sustained even longer in cells treated with 200 and 500 µM NO donors (**Figure 2A**). In addition to the A375 cells used in this time and concentration titration experiment, four other human melanoma cell lines were employed and exposed to varying concentrations of DETA NONOate for 2 h to examine pAktS473 protein levels as a measure of AKT activation. A similar increase of pAKT S473 was also observed in MeWo cells (BRAF and NRAS WT and PTEN intact) and LOX IMVI cells (BRAF V600E and PTEN intact). Conversely, two human melanoma cell lines without PTEN expression, WM 266-4 and WM 793, constitutively expressed the expected high basal pAktS473 level, and those levels were not further increased with the same NO exposure (**Figure 2B**). Therefore, only in cell lines with intact PTEN was NO able to drive increased AKT activity, supporting a direct involvement of NO with functional wildtype PTEN inactivation.

Akt activation depends on upstream PI3K activity, which produces the signaling intermediate PIP_3_ that brings AKT to the membrane. PTEN inhibits AKT activation by dephosphorylating phosphatidylinositols at the 3’-position. To test whether the NO-induced activation of Akt is PI3K-dependent, we applied two different PI3K inhibitors (LY 294002; Wortmannin) to A375 cells before NO exposure. Both PI3K inhibitors eliminated basal phospho-AKT and prevented NO-induced AKT activation (**Figure 2C**).

Together, these results indicate that NO stimulates AKT activation in some melanoma cells through a PI3K-dependent manner and suggest that a functional (and intact) PTEN is necessary for this effect.

### NO Stimulated AKT Activation Is Functional and Results in AKT Pathway Target Phosphorylation

**Figure 3:**
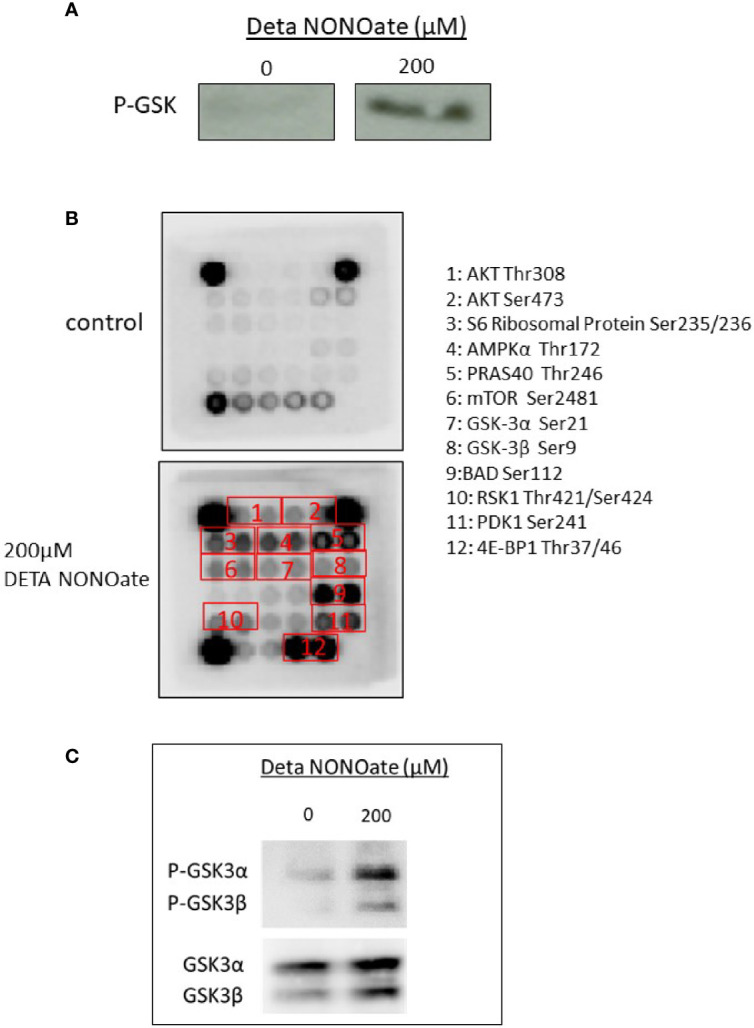
AKT activation increases phosphorylation in the AKT pathway targets. **(A)** AKT kinase activity was measured by AKT immunoprecipitation from cell extracts, followed by an *in vitro* kinase assay using GSK-3 fusion protein as a substrate. Phosphorylated GSK-3 levels in A375 cells treated with 200 μM DETA NONOate for 2 h. were measured by Western blotting. **(B)** Phosphorylation of AKT pathway targets after 2 h. of treatment with 200 µM DETA NONOate. The protein phosphorylation of 16 AKT pathway targets was examined using the antibody assay kit. Of the 16 AKT pathway targets, 12 showed increased phosphorylation after DETA NONOate treatment. **(C)** Western blotting was used to validate the GSK-3α and GSK-3β phosphorylations in A375 cells treated with 200 μM DETA NONOate for 2 h.

As pAKT S473 levels are a surrogate for Akt activation, we further directly measured AKT kinase activity *in vitro*. A375 cells were exposed to 200 µM DETA NONOate for 2 h, then AKT was immunoprecipitated to measure its kinase activity using GSK-3 fusion protein as the substrate. As shown in **Figure 3A**, AKT from NO treated cells is able to form phosphorylated GSK-3 as detected by western blot, as compared to the negligible level of untreated A375 cells. This confirms that NO stimulates AKT kinase activity.

AKT is a key kinase that can phosphorylate over 100 downstream substrates involved in diverse cellular functions including growth, survival, proliferation and metabolism (14). To screen for activation of such additional substrates, we used a commercial AKT pathway substrate antibody array, and found that out of the 16 Akt pathway targets included in the array, 12 targets had increased phosphorylation in the lysates from A375 cells after exposure to NO (**Figure 3B**). These targets including GSK-3, S6 ribosomal protein, PRAS 40, and 4E-BP1. Selected protein phosphorylation was also validated by western blot in A375 cells treated with DETA NONOate (**Figure 3C**). These results collectively reveal that NO-induced AKT phosphorylation results in enhanced AKT activity and activation of multiple AKT pathway downstream effectors that mediate many of the oncogenic effects of this signaling pathway.

### PTEN Phosphatase Activity Is Attenuated, and PTEN is Nitrosylated to Form PTEN-SNO by Either Exogenous or Endogenous NO

**Figure 4:**
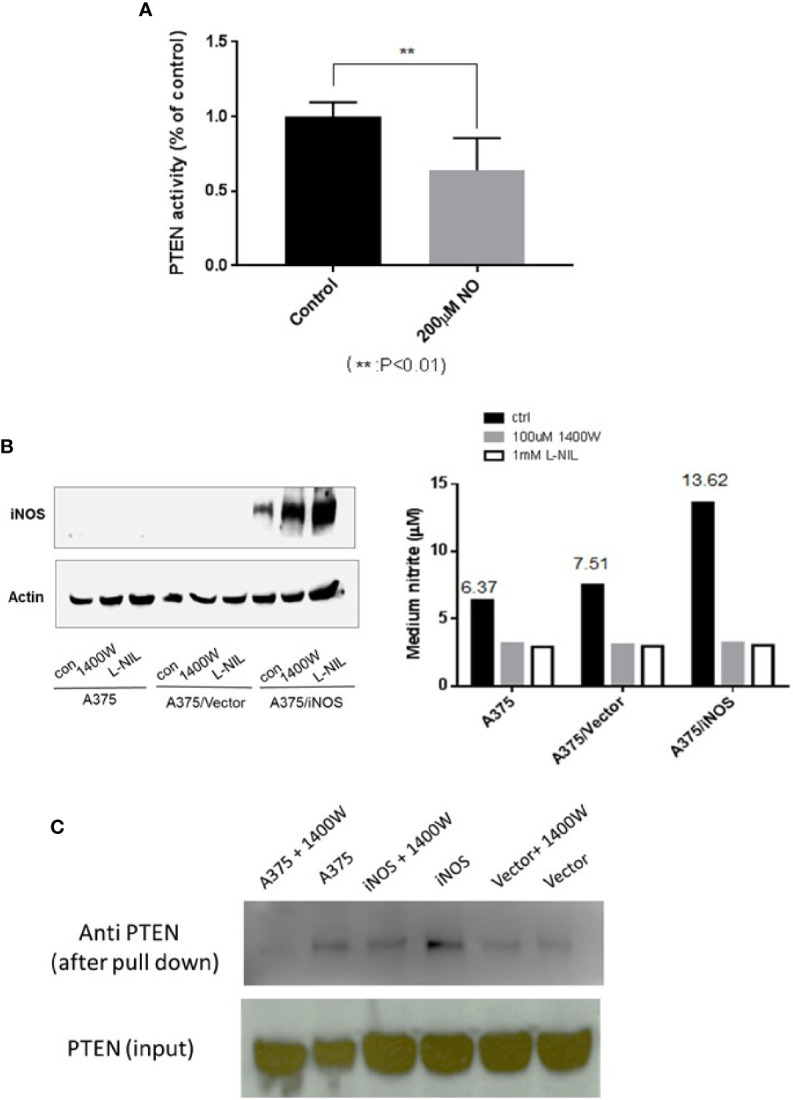
PTEN phosphatase activity is attenuated and NO induced PTEN–SNO formation. **(A)** PTEN phosphatase activity in A375 cells treated with 200µM DETA NONOate was measured with a malachite green assay. **(B)** A375 cells were transfected to express iNOS and produce endogenous NO. Transfected iNOS was confirmed with western blotting, and nitrite formation was measured with the Griess reaction. NO generation from iNOS was inhabitable with the NOS inhibitors L-NIL or 1400W. **(C)** Using a biotin-switch assay, we detected PTEN S-nitrosylation in A375 cells expressing iNOS.

PTEN antagonizes PI3K-AKT pathway activity through its lipid phosphatase activity. As NO treatment resulted in increased AKT activity, we tested if this treatment also affected PTEN’s lipid phosphatase activity. Thus, the phosphatase activity of PTEN, from A375 cells after treatment with DETA NONOate, was measured and compared with controls. The results demonstrate that NO treatment significantly decreased the lipid phosphatase activity of PTEN (**Figure 4A**). To further mimic the effects of endogenous NO produced by iNOS in cancer cells, human iNOS was transfected and expressed in A375 cells. iNOS transfection was validated by western blots of iNOS signal and the increase of nitrite production. In addition, iNOS inhibitors L-NIL and 1400W were utilized to inhibit iNOS activity and prevent it from producing NO as a control (**Figure 4B**). We then used the biotin-switch assay to confirm that the formation of PTEN-SNO in iNOS expressed A375 cells. Both the increased nitrite levels and PTEN-SNO were dependent on active iNOS, which was abolished when iNOS was inhibited by 1400W (**Figure 4C**).

## Discussion

Our combined analysis of clinical melanoma samples and human cell lines provides new insights into the role and clinical significance of iNOS expression and NO production in melanoma. While previous studies have implicated loss of PTEN expression as a significant event in melanoma, our data supports that post-translational modification of the intact PTEN protein by NO is another likely mechanism of tumor suppressor inactivation, which may also play an important role in this disease. In essence, our studies demonstrate that NO can inhibit PTEN activity and activate the PI3K-AKT pathway in melanomas with intact PTEN. As activation of the PI3K-AKT pathway has been implicated in resistance to both targeted and immune therapies, these results provide further evidence for a significant role for inflammation-driven nitric oxide, and indicate that such inflammation could be considered as a potential target in this disease (9).

Expression of iNOS in tumor cells, and a correlation between iNOS expression and poor clinical outcomes, have been found in many cancer types, including melanoma, breast, and colon, gastric and ovarian cancers (15). It is suggested that iNOS could be employed in conjunction with other tumor biomarkers to be better used for diagnostic purposes (16). Now our results indicate that the iNOS product, NO, functionally inactivates PTEN protein, contributing to its pro-oncogenic effects. It is well known that the normal tumor suppressor function of PTEN is lost if the gene acquires certain mutations, or is deleted. The inactivation of PTEN results in activating the PI3K-AKT pathway in melanoma and contributes to metastasis and therapeutic resistance. However, this only occurs in a minority of patients. For most melanoma patients with intact and potentially functional PTEN, our data suggests that expression of iNOS promotes AKT activation and is associated with worse survival in stage III disease patients. Notably, our analysis of the TCGA and stage III patients showed that iNOS is not associated with AKT activation or clinical outcomes in melanomas with loss of PTEN, likely due to the fact that iNOS appears to regulate the PI3K-AKT pathway through the formation of PTEN-SNO and inhibition of the lipid phosphatase activity of PTEN. Thus, our results suggest that the development of iNOS inhibition as a therapeutic strategy should focus on patients with intact PTEN.

In this study, we, for the first time, demonstrate the evidence of PTEN-SNO formation in human melanoma cells expressing iNOS. Protein S-nitrosylation (SNO) has emerged as an important post-translational modification; previously SNO sites have been reported on more than 4000 sites in over 2000 proteins (17). However, in any particular tumor within a range of NO flux is likely evident and dynamic. Therefore determining the SNOed protein abundance and ratio to that protein abundance is more important in determining the extent of functional consequence of protein SNO. Therefore, we are also working on using a novel saturation fluorescence thiol labeling approach (SNOFlo) to identify S-nitrosoproteome and quantitatively analyze the SNO level in melanoma cells to reveal the pathologically relevant protein candidates (18). The PTEN-SNO is one example of what may be the biochemical mechanism of inflammation driving cell survival, as SNO_PTEN was also found to attenuate neuronal survival in Parkinson’s disease (7). More recently, it is shown to initiate pro survival signaling of PI3K-AKT by PTEN-SNO and prevent neurons from degenerative death (19). Similarly, the same pro-survival mechanisms will benefit cancer cells and contribute to poor patient outcomes (20).

In addition to attenuated PTEN function, we attributed elevated AKT activity to the direct stimulation of PI3K-AKT by NO which is consistent with previous reports that concluded relative short lifetime of active and phosphorylated AKT (21) as Phosphorylated substrates are evident up to 2 h post-stimulation (22). Currently, phosphorylation of S473 is also known to occur through either mTORC2 (23) or DNA-PK (24). mTORC2 has been reported to be involved in NO-induced AKT phosphorylation in retinal cells (25) and NO has also been shown to upregulate the expression of DNA-PKs in tumor cells (26). Specifically, how NO triggers the activation of PI3K remains elusive; both cGMP-dependent and cGMP-independent mechanisms have been suggested. It was shown that in human T cells, NO led to PI3K recruitment to the effector domain of Ras, where it became activated *via* a process mediated by Ras Cys ^118^ redox modification (27). At the same time, the PI3K activation could also be induced by cGMP analog (8-bromo-cGMP) and can be blocked by ODQ, an sGC inhibitor indicating a cGMP dependent manner (28). It is likely that the cell types (malignant cells vs. normal cells) and the specifics of NO exposure (time, concentration, NO donor type) result in these differences. Our most recent publication on the role of this pathway in clinical samples reported that the tumor cell nitrotyrosine (NT) expression predicts a greater risk of developing CNS metastasis in those patients (29). Today, we understand that complex cancer cell and their response in the chronic inflammation environment guides us to develop better treatments for advanced melanoma.

NO can be produced both in tumor cells as well as other cells within the tumor microenvironment. Determined by its unique chemical property, NO reacts with many sites on intracellular molecules and hence affects a plethora of oncogenic pathways, such as tumor suppressor inactivation as suggested for PTEN in these studies, supporting consideration of NOS/NO targeted anti-cancer therapeutics (30). In summary, our study reveals that at tumor iNOS-relevant NO flux concentrations, NO can stimulate the PI3K-AKT pathway in human melanoma cells; identical conditions also promote NO-mediated PTEN-SNO. This experimental discovery is supported by clinical association data and has provided one plausible mechanistic insight into the possible functional consequences of inflammatory NO produced in human melanoma cells and their microenvironment, and possibly other iNOS expressing a subset of other cancers.

## Data Availability Statement

The raw data supporting the conclusions of this article will be made available by the authors, without undue reservation.

## Ethics Statement

The studies involving human participants were reviewed and approved by the institutional review boards of MD Anderson Cancer Center. The patients/participants provided their written informed consent to participate in this study.

## Author Contributions

All the authors contributed in designing the studies, executing the experiments, analyzing the data, writing the manuscript, and reviewing/editing. All authors contributed to the article and approved the submitted version.

## Funding

This work was supported by The University of Texas, MD Anderson Cancer SPORE in Melanoma (P50-CA093459) and MD Anderson Cancer Center Support Grant P30-CA016672, Foundation for the National Institutes of Health (FNIH)-PACT, Dr. Miriam and Sheldon G. Adelson Medical Research Foundation, Jim Mulva Foundation, and the AIM at Melanoma Foundations. Scientific and financial support for the PACT project are made possible through funding support provided to the FNIH by AbbVie Inc., Amgen Inc., Boehringer-Ingelheim Pharma GmbH & Co. KG, Bristol-Myers Squibb, Celgene Corporation, Genentech Inc., Gilead, GlaxoSmithKline plc, Janssen Pharmaceutical Companies of Johnson & Johnson, Novartis Institutes for Biomedical Research, Pfizer Inc., and Sanofi.

## Conflict of Interest

The authors declare that the research was conducted in the absence of any commercial or financial relationships that could be construed as a potential conflict of interest.

## Supplementary Material

Supplementary Figure 1 Sample Tumor iNOS and PTEN expression characteristics by immunohistochemical analyses. Stage III melanoma tissues from patients were immunostained for iNOS and PTEN, Scale 10X. As published previously (8), both intensity and percentage of positive cells were quantified and protein expression associating with overall survival (OS) in this retrospective tissue microarray.
