# Supplementary figures and images for: iNOS Associates With Poor Survival in Melanoma: A Role for Nitric Oxide in the PI3K-AKT Pathway Stimulation and PTEN S-Nitrosylation
Source: Front Oncol. 2021 Feb 12;11:631766. doi: 10.3389/fonc.2021.631766 (PMC7907506; doi:10.3389/fonc.2021.631766)

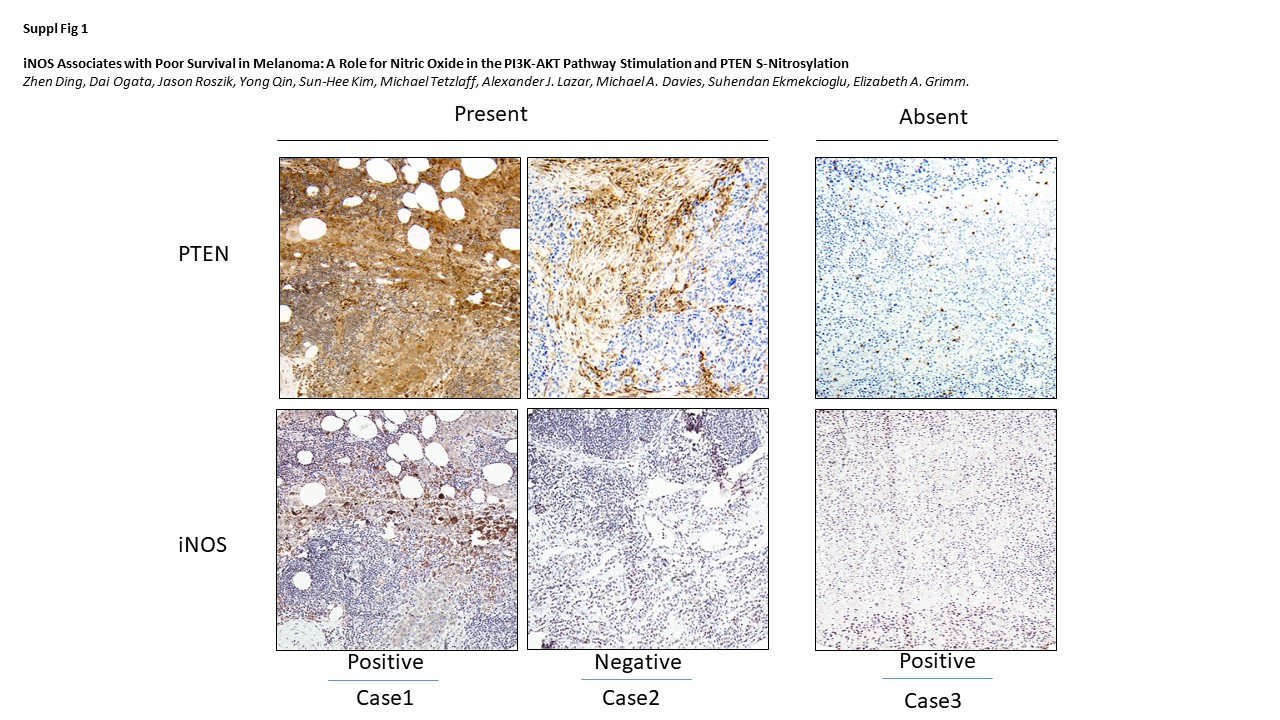

Supplement: Supplementary Figure 1 — Sample Tumor iNOS and PTEN expression characteristics by immunohistochemical analyses. Stage III melanoma tissues from patients were immunostained for iNOS and PTEN, Scale 10X. As published previously (8), both intensity and percentage of positive cells were quantified and protein expression associating with overall survival (OS) in this retrospective tissue microarray. [file Image_1.jpeg]
